# RP105-Negative B Cells in Systemic Lupus Erythematosus

**DOI:** 10.1155/2012/259186

**Published:** 2011-09-15

**Authors:** Syuichi Koarada, Yoshifumi Tada

**Affiliations:** Division of Rheumatology, Faculty of Medicine, Saga University, 5-1-1 Nabeshima, Saga 849-8501, Japan

## Abstract

Systemic lupus erythematosus (SLE) is a multisystem disease characterized by B cells producing autoantibodies against nuclear proteins and DNA, especially anti-double-strand DNA (dsDNA) antibodies. RP105 (CD180), the toll-like receptor- (TLR-) associated molecule, is expressed on normal B cells. However, RP105-negative B cells increase in peripheral blood from patients with active SLE. RP105 may regulate B-cell activation, and RP105-negative B cells produce autoantibodies and take part in pathophysiology of SLE. It is possible that targeting RP105-negative B cells is one of the treatments of SLE. In this paper, we discuss the RP105 biology and clinical significance in SLE.

## 1. Introduction

Systemic lupus erythematosus (SLE) is a prototypical multisystem disease characterized by dysfunction of T cells and polyclonal B-cell activation [1–4]. Although the pathogenesis of the autoimmune mechanisms in SLE is not fully understood, B cells producing autoantibodies against nuclear proteins and DNA, especially anti-double-strand DNA (dsDNA) antibodies, may be responsible for tissue damages as effector cells [5].

To date, the effective methods of target therapy for autoantibody-producing B cells for itself have not been established yet. RP105 (CD180), the toll-like receptor- (TLR-) associated molecule, is expressed on normal B cells [6]. We reported that RP105-negative B cells, that lacked RP105 expression on the surface, increase in peripheral blood from patients with active SLE [7]. It was proved that RP105-negative B cells produce autoantibodies, including anti-ds-DNA antibodies, in vitro [8]. 

These results suggest that RP105 may function as a negative regulator of B-cell activation. Recently, it was proved that RP105 is a negative regulator for TLR-4 in immune reactions in dendritic cells (DCs) [9, 10]. RP105 also regulates TLR-8 and TLR-9 responses and functions to limit autoreactive B-cell activation [11]. Moreover, it is possible that in the condition of high levels of BAFF (B cell-activating factor belonging to the TNF family) and APRIL (a proliferation-inducing ligand), RP105-negative B cells maintain autoreactivity in autoimmune diseases through BCMA (B-cell maturation antigen). Therefore, RP105-negative B cells are one of the target cells for treatment of SLE. In this paper, we discuss the RP105 biology and clinical significance in SLE.

## 2. RP105 (CD180) Biology

A type I transmembrane receptor, RP105 (radio protective MW105), was originally discovered as a surface marker of B cells in mice and humans [6, 12]. However, the molecules also express on monocytes [7], macrophages, and dendritic cells. RP105 has leucine-rich repeat (LRR) motifs, similar to the other TLRs with extracellular LRRs. The LRR is a motif involved in protein-protein interaction [13]. The molecules having LRRs take part in the recognition of exogenous pathogens and subsequent activation of the immune system in diverse species [14, 15]. The TLR was first identified in Drosophila [16] and has a function of triggering innate defenses against bacterial and fungal pathogens. In humans, LRR molecules are also important in the defense against pathogens. 

The fact of structural similarity of the extracellular domain of RP105 to TLRs suggests that RP105 senses pathogen invasion [17]. RP105 ligation by anti-RP105 antibodies transmits an activation signal leading to B-cell proliferation, resistance against radio-induced apoptosis, and costimulatory molecule expression of CD86, in mice [6]. However, RP105 lacks a conserved intracellular signaling domain (Toll-IL-1 receptor domain; TIR domain) and has a very short cytoplasmic tail, 11-amino-acids [18]. RP105 forms a complex with soluble protein MD-1, making heterodimer complex on surface of B cells [6, 17, 19]. MD-1 is essential for expression of RP105 on cell surface, similar to MD-2 for TLR-4. Although signaling molecules binding to tail of RP105 have not been identified yet, RP105 ligation triggers Lyn, Bruton tyrosine kinase (BTK), mitogen-activated protein kinase (MAPK), and NF-kB activation. The results of RP105- and MD-1-deficient mice showed that activation of B cells, including antibody production, CD86 expression, and proliferative response to LPS, was reduced [19].

On the other hand, it have been recently reported that RP105 has a negative regulatory function for TLR-4/MD-2 signal [9, 10]. We also investigated the role of RP105 in the development of collagen-induced arthritis (CIA). RP105-deficient mice accelerated the onset of arthritis and increased severity. The results indicate that RP105 regulates the antigen-presenting cell function and Treg development [10]. Interestingly, B cells activated with anti-RP105 antibodies show growth arrest and apoptosis upon antigen receptor signaling [20].

Collectively, in mice, RP105 may be considered to regulate the growth and death of B cells. However, in human, it has not been clarified yet which function is predominant. The information of RP105 obtained mainly from mice; there had been little information in human. Therefore, we started the study of RP105 in human SLE, in which autoantibody-producing and activated B cells play pivotal roles.

## 3. RP105 in SLE

SLE is a typical multisystem disease of unknown etiology [21] characterized by dysfunction of T cells and B cells. Together with the production of autoantibodies against nuclear proteins and DNA, polyclonal B-cell activation is one of outstanding features of SLE [21]. Therefore, we had estimated that B cells producing autoantibodies, which avoid apoptosis and maintain their activated state in peripheral blood, may have abnormal expression of RP105.

In contrast to murine RP105, little was known about the molecule in humans. The human homologue of RP105 has been identified and its mAb also established in 1998 [12, 22]. Therefore, it is important to clarify the role of RP105 on B cells in human SLE. Staining of PBMCs from SLE patients and normal subjects was performed with anti-human CD19 and RP105 antibodies, and analyzed using FACScan. It has been shown that RP105 is expressed on virtually all mature B cells from normal subjects. Interestingly, however, we found that the number of RP105-negative B cells was dramatically increased in SLE patients [7].

It has been also suggested that the disease activity of SLE, SLE-DAI scores, was related to the number of RP105-negative B cells. Serial changes of RP105-negative B cells from active SLE patients were analyzed individually after treatment. The percentages of RP105-negative B cells decreased as the disease turned inactive. To know B cell function, serum levels of IgG were measured. The levels were correlated with the percentage of RP105-negative B cells. These results suggest that the emergence of RP105-negative B cells in the peripheral blood closely relates to the disease activity and B-cell function of SLE.

RP105-negative B cells disappeared in the peripheral blood from inactive patients treated with corticosteroids [2]. It is estimated that RP105-negative B cells seem to be more sensitive to corticosteroids than RP105-positive B cells [2]. The effects of dexamethasone on the apoptosis of RP105-negative B cells in vitro were examined. RP105-negative B cells underwent spontaneous apoptosis, compared to RP105-positive B cells. Dexamethasone induced apoptosis of RP105-negative B cells, although in contrast, apoptosis of RP105-positive B cells were not induced. This result may illustrate in vivo phenomena, clearance of RP105-negative B cells, in patients after treatment with corticoids [2].

In patients with SLE, ANA in serum is generally positive. Therefore, ANA-negative SLE is very rare [23]. However, ANA-negative SLE seems to be a subpopulation of SLE, and the diagnosis may be difficult in patient without immunological disorders [24–31]. We present that RP105-negative B cells were increased in the peripheral blood of two patients with ANA-negative SLE [32]. The numbers of RP105-negative B cells were associated with disease activity even in ANA-negative SLE patients [32]. Without significant serological markers for SLE, examination of RP105 on B cells may be useful in evaluation of activity. Later, the patients turned out to be serologically positive, including ANA, dsDNA, and anti-Sm antibody.

## 4. Pathophysiology of RP105-Negative B Cells in Autoimmune Diseases

The most important issue was to clarify the functional roles of RP105-negative B cells in SLE. RP105-negative B cells and -positive B cells from patients with SLE were separated using a cell-sorter and analyzed production of IgG and IgM antibodies in vitro. Spontaneous IgG and IgM antibodies from RP105-negative B cells in vitro were enhanced by a mitogen, staphylococcus aureus Cowan I (SAC), or IL-6. Surprisingly, RP105-negative B cells but not RP105-positive B cells produced IgM and IgG class anti-dsDNA antibodies in the culture with activated T cells by anti-CD3 antibodies and IL-10 [8]. These results suggest at least partially that the population of RP105-negative B cells may include pathogenic autoreactive B cells subset(s).

It has been shown that B cells from RP105-deficient mice were hyporesponsive to TLR-4 and TLR-2 stimulation [33, 34], Divanovic et al. showed that RP105 directly interacts with TLR-4 and negatively regulates TLR-4 signaling by experiments using transfectant cells and RP105-deficient mice-derived DCs [9]. These results are suggestive to consider the function of RP105 in human SLE. It is possible that loss of RP105 induced the dysregulation of TLRs and maintain autoreactive response of B-cell hyperactivation and production of auto-antibodies and result in autoimmunity, ultimately.

## 5. Characterization and Phenotypes of RP105-Negative B Cells

 To characterize RP105-negative B cells in SLE patients, a multicolor analysis was performed using a flow cytometry [7]. RP105-negative B cells expressed higher levels of CD95 and CD86 but not CD80. RP105-positive B cells showed no expression of these molecules. Although RP105-positive B cells expressed low levels of CD38, RP105-negative B cells expressed higher levels of CD38. Surface IgD and IgM levels were low on RP105-negative B cells; however, RP105-negative B cells lacked intracellular IgM but about a half of the RP105-negative B cells had intracellular IgG. RP105-negative B cells can produce class-switched Ig (IgG) although it is still stored in the cytoplasm of B cells. RP105-negative B cells were distinct from CD5-positive cells, since almost all RP105-negative B cells were negative for CD5. Moreover, RP105-negative B cells are CD20-negative and CD28-positive. Therefore, RP105-negative B cells may belong to late B cells. Also CD138 expression was dull (submitted). The phenotype of RP105-negative B cells was summarized as CD95-positive, CD86-positive, CD38-bright, IgD-negative, IgM-dull, intracellular Ig-positive, large sized B cells, which is consistent with the phenotype of activated and effector B cells differentiated into plasma cells. However, so far, this phenotype of B cells had not been reported. RP105-negative B cells may consist of subsets between early plasmablasts and plasma cells. However, another possibility that RP105-negative B cells emerge only in autoimmunological pathogenic state could be proposed.

## 6. RP105-Negative B Cells in Various Rheumatic Diseases

The expression of RP105 on peripheral blood B cells from patients with various types of rheumatic diseases was examined by a flow cytometer [35]. The percentages of RP105-negative B cells varied among the diseases. The numbers of RP105-negative B cells in the peripheral blood of patients with Sjogren's syndrome (SS) and dermatomyositis (DM) were increased. In other rheumatic diseases, including rheumatoid arthritis (RA), systemic sclerosis (SSc), angiitis syndrome, Behçet's disease, mixed connective tissue disease (MCTD), and polymyositis (PM), the numbers of RP105-negative B cells were slightly increased compared with normal subjects but the levels were low. 

Although, clinically, DM and PM (except for the presence of skin manifestations) are similar, two diseases are etiologically different [36–38]. The involvement of humoral immune mechanisms in DM and cellular immunity in PM are proposed. The levels of RP105-negative B cells in the peripheral blood of active patients with DM and PM were analyzed by flow cytometry [39]. The percentage of RP105-negative B cells in PM was low, and increased RP105-negative B cells were found in DM. Interestingly, bronchoalveolar lavage fluid from a DM patient contained a large number of RP105-negative B cells. The increase of RP105-negative B cells is a marker of DM, and B cell activation in DM but in PM seems to be pathophysiologically different.

 Because it was well established that B cells from SS patients are in a polyclonally activated state [40], it is conceivable that a large proportion of the RP105-negative B cells existed in SS patients [41]. RP105-negative B cells from SS patients produced IgG and IgM spontaneously in vitro. The production of Ig was enhanced by SAC or IL-6. Salivary glands from some SS patients were found to have lymphoid follicles whose germinal centers consisted of RP105-negative B cells. A larger proportion of B cells infiltrating the area other than lymphoid follicles was also negative for RP105. RP105-negative B cells may be associated with the inflammation and tissue damage of the salivary glands.

We have demonstrated that the RP105-negative B cells were significantly increased in SLE, SS, and DM in which B cell activation is postulated to be involved. Also, the RP105-negative B cells were located in the impaired organs, such as lung or salivary gland and related to autoimmunological inflammation. These results suggest that RP105-negative B cells may be one of the targets of treatment with refractory autoimmune diseases with organ involvements.

## 7. Future Biologic Agents in the Treatment of SLE, Targeting RP105-Negative B Cells

For treatment of SLE, one of the targeting B cell therapies is rituximab (anti-CD20 antibodies) [42], which has shown effective response in SLE and RA patients. If possible, the most promising therapy of SLE may be a strategy of targeting autoantibody-producing B cells. Because RP105-negative B cells produce anti-dsDNA antibodies, the therapy of targeting RP105-negative B cells may be one of the possible therapies for SLE. For this purpose, we are studying the identification of the antigens specific for RP105-negative B cells using DNA microarrays [43]. Differential gene expression between RP105-negative and RP105-positive B cells was analyzed and surface expression of antigens specific for RP105-negative B cells was confirmed by a flow cytometry. Although several antigens were identified, BCMA was one of the most interesting antigens. RP105-negative B cells from SLE patients showed more preferential expression of BCMA compared with BAFF-R than normal subjects and were possibly regulated by BAFF/APRIL. These results also suggest that BCMA may be one of the target for elimination of RP105-negative B cells to treat SLE. The data of BCMA expression suggest that RP105-negative B cells from active SLE patients may have predisposition toward survival response in high levels of BAFF and/or APRIL in vivo via BCMA. 

Because the nonspecific immunosuppressive therapies induce significant adverse effects and disability in autoimmune diseases, novel safer and more effective therapies specific for pathogenic cells or molecules are required. Our results suggest that blocking agents to the signals between BAFF/APRIL and their receptors, especially the passage via BCMA may be effective. Prevention of emerging or depletion of RP105-negative B cells may be an additional option for the treatment of SLE patients. Further studies will be required to determine whether targeting RP105-negative B cells could inhibit autoantibody production and regulate autoimmune events. Our results in human SLE would provide a new insight of potential mechanism and intervention of B cells in autoimmune diseases.

## 8. Conclusions

RP105 may regulate B-cell activation, and RP105-negative B cells produce autoantibodies and take part in pathophysiology of SLE. Autoantibody-producing RP105-negative B cells are considered as one of the therapeutic targets in SLE. Moreover, the targeting RP105-negaive B cells would be a specific treatment for pathogenic cells, and the efficacy and safety should be confirmed in further studies.
